# Effects of Process Variables on the Physicochemical, Textural, and Structural Properties of an Isolated Pea Protein-Based High-Moisture Meat Analog

**DOI:** 10.3390/foods12244413

**Published:** 2023-12-08

**Authors:** Yu Zhang, Gi Hyung Ryu

**Affiliations:** Department of Food Science and Technology, Food and Feed Extrusion Research Center, Kongju National University, Yesan 32439, Chungnam, Republic of Korea; d20210174@smail.kongju.ac.kr

**Keywords:** isolated pea protein, high-moisture meat analog, process variables, physicochemical properties, textural properties

## Abstract

This study investigated the optimal extrusion conditions required to produce an isolated pea protein (IPP)-based meat analog. High-moisture extrusion cooking (HMEC) was performed. The effects of the moisture content (55 and 60%), barrel temperature (165 and 175 °C), and screw speed (150 and 200 rpm) on the physicochemical, textural, and structural properties of the high-moisture meat analog (HMMA) were determined. The results showed that the moisture content had a significant effect (*p* < 0.05) on the physicochemical and textural properties of the HMMA. A lower moisture content had significant impact (*p* < 0.05) on enhancing the texturization of the HMMA and the formation of fibrous structures, thereby increasing the texture profile analysis (TPA) and cutting strength of the HMMA. Protein denaturation during HMEC resulted in a lower protein solubility of the meat analog than the raw material. The content of *β*-sheets and *β*-turns in the meat analogs were higher than that in the raw material, while the content of random coils and *α*-helices is inversely proportional. The process variables had no significant (*p* > 0.05) effect on the secondary structures. In conclusion, the moisture content is the most important factor affecting the properties of HMMAs. The extrusion process variables for HMMAs are a moisture content of 55%, a barrel temperature of 175 °C, and a screw speed of 200 rpm.

## 1. Introduction

Excessive meat consumption has been linked to various human health issues, environmental problems, and the safety of animal products [1]. As a result, there is a growing interest in developing meat analogs to provide an alternative source of protein and overcome such environmental impacts. Meat analogs are food products made from plant proteins that resemble real meat in terms of texture, flavor, and appearance [2]. The findings of Michcel et al. [3] demonstrate that meat alternatives have the best chance of successfully replacing meat when they closely resemble highly processed meat products in terms of their taste and texture and are offered at competitive prices. Various meat analog products are currently available, including chicken-like blocks, ground beef-like products, nuggets, steaks, burgers, and sausages [4]. Soybeans, peas, and wheat gluten have been used as sustainable protein sources for producing meat analogs, while rice, peanuts, mung beans, and corn starch have also been utilized [5,6].

Peas (*Pisum sativum* L.) are a valuable source of high-quality plant protein in the human diet, which typically contains 20–30% proteins (consisting of 65–80% globulins and 10–20% albumins) [7]. Isolated pea proteins (IPPs) are high-protein, low-carbohydrate, and low-fat powders made by extracting protein from peas [8]. IPP has been widely used as a plant protein source in meat analogs as it is nutritious, inexpensive, and non-allergenic [9]. In addition, IPP possesses antioxidant activities, antihypertensive effects, and the ability to modulate gut microbiome activities [10]. The finding of Samard and Ryu [11] indicated that IPP-based meat analogs had a high water-holding capacity, integrity index, and textural properties, and that the number of sulfur-containing amino acids increased after extrusion, suggesting that IPP-based meat analogs have a high potential for physicochemical and functional properties in high-moisture meat analogs (HMMAs). Schreuders et al. [12] reported that fibrous meat analogs with a comparable matrix strength to cooked chicken meat were produced using a blend of IPP and wheat gluten. Wheat gluten (WG) is typically a by-product produced during the separation of starch from wheat flour, known for its good viscoelasticity, extensibility, and thermal coagulation properties. Moreover, WG can be readily transformed into fibrous alignment materials [4]. Studies suggest that when soybean proteins like ISP, SPC, or FFS are added to WG, the fiber alignment and layering increase, addressing the texture deficiencies in soy-based materials [13,14,15]. Corn starch (CS) is commonly used in the base material of extruded meat analogs as a binding agent because it enhances the cohesion among protein molecules.

Extrusion is an effective and straightforward technique for producing meat analogs. The process can be classified into low-moisture extrusion cooking (LMEC) (moisture content: 20–40%) and high-moisture extrusion cooking (HMEC) (moisture content: 50–80%) with a cooling die. Compared to LMEC, HMEC has become preferable in producing meat analogs with fibrous structures and meat-like textures. The HMEC process allows the denaturation, rearrangement, and phase transition of protein molecules to occur at high temperatures, pressure, and shear. Then, the fibrous or layered fibrous structure of proteins is obtained once the immobilization takes place with cooling [16,17,18]. The properties of meat analogs are primarily dependent on the composition of protein sources and the extrusion process parameters, such as the moisture content, barrel temperature, and screw speed [17,19]. The composition of protein sources indicates the final structure morphology, such as gel, layered, and fibrous structures of meat analogs. Extrusion process parameters play an important role in forming texture and structure [20]. It has been reported that moisture content is the most critical factor for product texturing characteristics, while the barrel temperature and screw speed are important for texturization [21]. For instance, Xiao et al. [22] also revealed that the increase in moisture content reduces the hardness and tensile strength of the rice bran-based meat analog. In addition, Lin et al. [23] reported that the structure of the products became more directionally aligned with decreasing extruded moisture content. Meanwhile, the extrusion temperature is a key factor affecting the conformational changes of the protein, which, in turn, will further affect the final quality of the organized plant protein [24]. Higher screw speeds promote the dispersion of the dispersed phase (mainly carbohydrates, etc.) in the continuous phase (proteins), which facilitates the formation of a finer fiber structure [25].

For decades, the effects of extrusion process parameters on the structure and function of several protein sources have been reported in the literature [19,23,26,27,28,29]. However, systematic studies investigating the effects of extrusion process variables on IPP-based meat analogs are lacking. The purpose of this study was, therefore, to optimize the extrusion process parameters, including the moisture content (55 and 60%), barrel temperature (165 and 175 °C), and screw speed (150 and 200 rpm), that are required to produce an IPP-based meat analog with a desirable fibrous structure and meat-like texture. The quality of the obtained meat analogs was determined through their physicochemical, textural, and structural properties.

## 2. Materials and Methods

### 2.1. Materials

This study used blends of isolated pea protein (IPP), wheat gluten (WG), and corn starch (CS) as plant-based protein sources. The IPP was purchased from Yantai Shuangta Food Co., Ltd. (Yantai, China). The WG and CS were purchased, respectively, from Roquette Frères (Lestrem, France) and Samyang Ltd. (Ulsan, Republic of Korea).

The optimum ratio of the raw material formulation was observed in the previous study as IPP/WG/CS = 5:4:1 *w*/*w*, since the obtained meat analog showed high texturization and a more fibrous structure [30].

### 2.2. Extrusion Process

**Figure 1:**
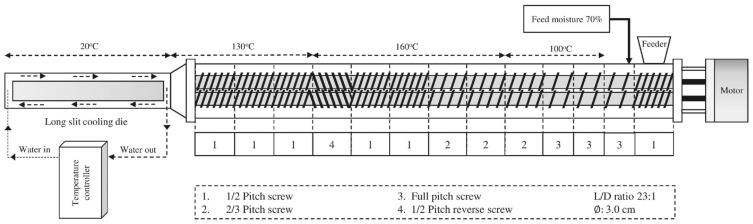
Schematic diagram of high-moisture extrusion cooking with details of the screw configuration.

The HMMAs were produced using a co-rotating intermeshing twin-screw extruder (Incheon Machinery Co., Ltd., Incheon, Republic of Korea) with a 3 cm diameter and 69 cm length (L/D ratio = 23), equipped with three heating zones and a long-slit cooling die (7 × 1 × 50 cm). Cold water (20 °C) was used to control the temperature of the extruded melt. The screw configuration is depicted in Figure 1. The feed rate was fixed at 100 g/min. The extrusion process was performed at various feed moisture contents (55 and 60%), barrel temperatures (165 and 175 °C), and screw speeds (150 and 200 rpm). The HMMAs were packed in sealed bags and stored at 4 °C until used. Parts of the HMMAs were freeze-dried, ground, and stored at room temperature.

### 2.3. Integrity Index

The integrity index indicates the percentage of meat analog residue after hydration, autoclaving, homogenization, and drying. The integrity index of the HMMA was determined according to the method described by Samard et al. [14], with slight modifications. The meat analogs were cut into 1 × 1 × 1 cm squares and mixed with 100 mL of distilled water in a conical flask. The samples were then placed in a water bath set at 90 °C for 30 min before autoclaving at 121 °C for 15 min (PAC-60, Lab House Co., Ltd., Seoul, Republic of Korea). The autoclaved samples were poured into a 20-mesh sieve, rinsed with running water for 1 min, and homogenized with a homogenizer (T10 Basic Ultra-Turrax, IKA Co., Ltd., Seoul, Republic of Korea) at 17,530× *g* for 1 min. Finally, the samples were rinsed on a 20-mesh sieve for 1 min under running water. The samples on sieves were dried at 105 °C until a constant weight was reached. The experiment was carried out in triplicate. The integrity index (g kg^−1^) was calculated using Equation (1).

Integrity index (g kg^−1^) = [(W_c_ − W_b_)/W_a_] × 1000, (1)

where W_a_ is the sample weight (dried base), W_b_ is the sieve weight, and W_c_ is the total weight of the dried sample and sieve.

### 2.4. Nitrogen Solubility Index (NSI)

The nitrogen solubility index refers to the soluble protein produced during the manufacture of meat analogs [20,30]. The NSI was measured according to the method of Samard et al. [14] with some modifications. In this study, the total nitrogen content was extracted by mixing the ground sample (0.2 g) with 6 N HCl (5 mL). The mixture was then hydrolyzed at 100 °C for 24 h before dissolving in 10 mL of distilled water. On the other hand, the soluble nitrogen content was extracted by mixing the ground sample (0.2 g) with 0.5% KOH (10 mL) prior to shaking at 120 rpm at 30 °C for 30 min using a shaker (H-1000-3, Hanil Science Industrial Co., Gangneung, Republic of Korea). Then, the total nitrogen content mixture and soluble nitrogen content mixture were centrifuged at 3000 rpm for 30 min. The supernatant (0.05 mL) was collected and boiled at 100 °C with a ninhydrin solution (1.67 mL) for 10 min. After dilution, the absorbance at 575 nm of the sample was determined using a spectrophotometer (UL 61010-1, MET Laboratories, Inc., Baltimore, MD, USA). Distilled water was used as a blank. The NSI expressed as g kg^−1^ was calculated using Equation (2).

NSI (g kg^−1^) = Soluble nitrogen content/Total nitrogen content × 1000, (2)

### 2.5. Protein Solubility

Hydrogen bonds, hydrophobic interactions, and disulfide bonds are the main forces that maintain the tertiary structure of proteins, whereas SDS, urea, and *β*-mercaptoethanol (*β*-Me) can disrupt these forces, respectively, leading to different protein solubility levels. The protein solubility of the meat analog was determined in comparison to blends of IPP, WG, and CS, following the method of Chiang et al. [26] with slight modifications. Briefly, samples (0.5 g) were orderly mixed with the following reagents (10 mL each): (1) 0.035 mol/L of phosphate buffer, pH 7.6 (P); (2) 1.5 g of sodium dodecyl sulfate (SDS) in 100 mL of phosphate buffer (P + S); (3) 8 M Urea in phosphate buffer (P + U); (4) 0.1 mol/L of *β*-Me in phosphate buffer (P + M); (5) P + S + U; (6) P + S + M; (7) P + U + M; and (8) P + S + U + M. The sample–reagents mixture was then shaken for 2 h to allow for the extraction of proteins. After centrifugation at 4000 rpm for 20 min, the supernatant was collected, and the residual protein content was measured using the Bradford method. The protein solubility (%) was calculated using Equation (3).

Protein solubility (%) = Soluble protein in the supernatant/Total protein in the samples ×100, (3)

### 2.6. Texture Profile Analysis (TPA), Cutting Strength, and Texturization Degree

TPA provides information on the behavior of meat analogs while mimicking the human biting activity through a double compression test. The cutting strength, in addition, indicates the resistance force once the products are cut. Texture parameters, including springiness (relating to the elasticity), cohesiveness (indicates the particle–particle agglomeration), and chewiness (indicates the desirable mouthfeel of a product before swallowing), as well as the vertical- and parallel-direction cutting strengths (indicates the strength of the sample fiber structure) and texturization degree of the HMMA were determined.

A TPA of the samples was performed under a Sun rheometer (Compac-100, Sun Science Co., Tokyo, Japan). The HMMAs (1 × 1 × 1 cm) were compressed using a cylindrical probe (25 mm diameter). The textural profile analysis parameters, including springiness, cohesiveness, and chewiness, were measured under the following conditions: a crosshead speed of 100 mm/min, a maximum peak stress of 10 kg, and a distance between the two holders of 20 mm. The vertical (F_v_) and parallel (F_p_) cutting strength of the samples (1 × 1 × 1.5 cm) was measured with a cutting probe (7.5 mm × 38.3 mm), using a maximum peak stress of 10 kg. The springiness, cohesiveness, hardness, and chewiness were calculated using Equations (4)–(6), respectively, according to Trinh et al. [31]. The texturization degree indicating fibrous structure formation was calculated as the ratio of F_v_ and F_p_ described by Maung et al. [21]. The results are reported as the average ± SD from 10 measurements.

Springiness (%) = D_2_/D_1_ × 100 (4)

where D_1_ is the original compression distance and D_2_ is the distance of the detected height during the second compression.

Cohesiveness (%) = A_2_/A_1_ × 100 (5)

where A_1_ is the area of work during the first compression and A_2_ is the area of work during the second compression.

Chewiness (N) = Springiness × Cohesiveness × H(6)

where H is the maximum force during first cycle of compression.

### 2.7. Scanning Electron Microscopy (SEM)

The microstructure of the samples was studied using an FE-SEM (MIRA III LMH, Tescan Co., Cranberry Township, PA, USA). The freeze-dried HMMA (2 × 2 × 2 mm) samples were fixed to the specimen holder, sputter-coated with a silver–palladium alloy, and subjected to the microscope at an accelerating voltage of 10 kV.

### 2.8. Fourier Transform Infrared Spectroscopy (FT-IR)

The secondary structure of the HMMA compared to raw material blends was studied using FT-IR at a wavenumber ranging from 4000 to 400 cm^−1^. The FT-IR spectra of the samples showed typical peaks at 1600–1640, 1640–1650, 1650–1658, and 1660–1700 cm^−1^, which are assigned to the protein structures of β-sheets, random coils, α-helices, and β-turns, respectively [32]. The ground sample (1 mg) was mixed with KBr (100 mg) before being pressed into transparent flakes. Pure KBr pellets were used as background scans. The infrared curves were baseline-corrected, normalized, and Fourier-deconvoluted using the OMNIC 9.2 software (Thermo Fisher Scientific Inc., Waltham, MA, USA). The percentage of the sub-peak structures in the amide I region (1600–1700 cm^−1^) was analyzed and calculated using the PeakFit v4.12 software (SeaSolve Software Inc., Framingham, MA, USA).

### 2.9. Experimental Design

The effects of the extrusion process parameters on the properties of the HMMA were studied through a 2^3^ full factorial experimental design. A one-way analysis of variance (ANOVA) was carried out for statistical analyses using the IBM SPSS software, version 22.0 (IBM, Armonk, NY, USA). Differences between means were compared using Duncan’s multiple range test at a significance level of *p* < 0.05.

## 3. Results and Discussion

### 3.1. Physicochemical Properties

#### 3.1.1. Integrity Index

Table 1 shows that the integrity index of the HMMAs was significantly affected (*p* < 0.001) by the moisture content, barrel temperature, screw speed, and their interactions. The highest to lowest influence was that of the barrel temperature, moisture content, and screw speed.

**Figure 2:**
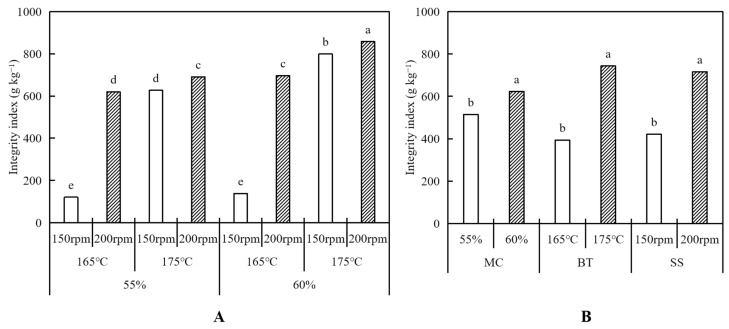
Integrity index of meat analogs with different process variables (moisture content—MC, barrel temperature—BT, screw speed—SS) (**A**). The average values of integrity index for samples, respectively (**B**). Bars with the same letter within the same textural property are not significantly different at *p* < 0.05 based on Duncan’s multiple range test.

The integrity index of the IPP-based HMMA was observed, ranging from 120.68 to 858.09 g kg^−1^ (Figure 2A), and it significantly (*p* < 0.001) increased as the moisture content increased (Figure 2B). This result is consistent with that of Cho and Ryu [27]. The reason might be because extrusion at a low moisture content generates weak and dispersed structures, resulting in a low integrity index of the product [27]. High barrel temperatures and fast screw speeds may also result in tough fibrous structures due to the rearrangement and cross-linking of protein molecules to form fibrous structures as the molten material passes through the long cooling die of the HMEC, thus increasing the integrity index of the HMMA after extrusion [14,18,30].

#### 3.1.2. Nitrogen Solubility Index

**Figure 3:**
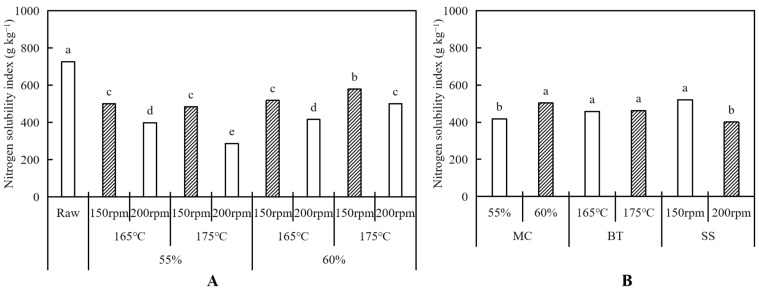
Nitrogen solubility index of meat analogs with different process variables (moisture content—MC, barrel temperature—BT, screw speed—SS (**A**). The average values of nitrogen solubility index for samples, respectively (**B**). Bars with the same letter within the same textural property are not significantly different at *p* < 0.05 based on Duncan’s multiple range test.

As shown in Figure 3A, the NSI values of the HMMA were observed to range from 287.11 to 578.58 g kg^−1^, significantly (*p* < 0.05) lower than that of raw protein blends (726.16 g kg^−1^). The loss of protein solubility increased the denaturation of the protein, resulting in a decrease in the NSI value after extrusion, which affected the texturization of the protein molecule. The NSI values were mainly affected by the moisture content and screw speed (Table 1). This finding agrees with the study of Wang et al. [33], indicating no significant correlation between the barrel temperature and the NSI value of the HMMA.

The NSI value increased with the increase in moisture content (Figure 3B). This can be explained by the fact that in HMEC, an increase in moisture content results in a lower temperature and pressure in the barrel, and therefore a lower degree of denaturation of the meat analog, less insoluble protein produced, and increased solubility [23,33]. The NSI value, on the other hand, decreased with the increasing screw speed since, when shear was applied, the friction between the raw material and the barrel wall and between the raw material and the screw increased and denaturation was further exacerbated, leading to extensive protein unfolding, the formation of large insoluble aggregates, and, ultimately, reduced solubility [34]. In this study, the lowest NSI value, which indicates the highest texturization of the HMMA, was obtained from the condition using a 55% moisture content, a 175 °C barrel temperature, and a 200 rpm screw speed (Figure 3A).

#### 3.1.3. Protein Solubility

**Figure 4:**
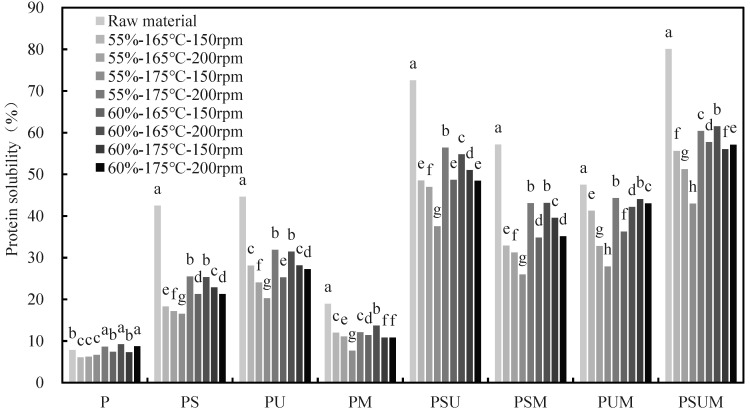
Protein solubility of natural IPP and HMMA in eight extractants. P, phosphate-buffered solution; S, sodium dodecyl sulfate; U, urea; M, *β*-mercaptoethanol. Bars with the same letter within the same textural property are not significantly different at *p* < 0.05 based on Duncan’s multiple range test.

As shown in Figure 4, the protein solubility levels of the HMMAs in all eight extraction solvents were lower than that of the raw material after extrusion, which suggests that the extrusion process affected protein aggregation. Zhang et al. [35] reported the same results, and they suggested that this phenomenon might be due to the formation of some new chemical bonds to maintain the protein structure during the extrusion process.

The significant increase in protein solubility after the disruption of hydrophobic interactions and hydrogen bonds by PS and PU solutions, respectively, suggests that hydrophobic interactions and hydrogen bonds are the major protein intermolecular interactions in IPP HMMAs. Hydrogen bonds are the main force that maintains the structure of the product during the texturization of high-moisture soybean protein, which Lin et al. [36] reported. In addition, the solubility of the HMMAs and raw materials in the PM solution is significantly lower than that of PM and PU, so we can infer that the major force to stabilize the protein structure is not attributed to the disulfide bonds. This result is comparable to that of extruded soy protein reported by Lin et al. [36] and pea protein reported by Zhang et al. [37]. Using the combination of all solvents, PSUM could solubilize the highest levels of proteins in the HMMA. These results indicate the existence of interactions between various chemical bonds [38]. However, the process variables had no regular effect on the HMMA’s protein solubility.

### 3.2. Textural Profile Analysis, Cutting Strength, and Texturization Degree

**Figure 5:**
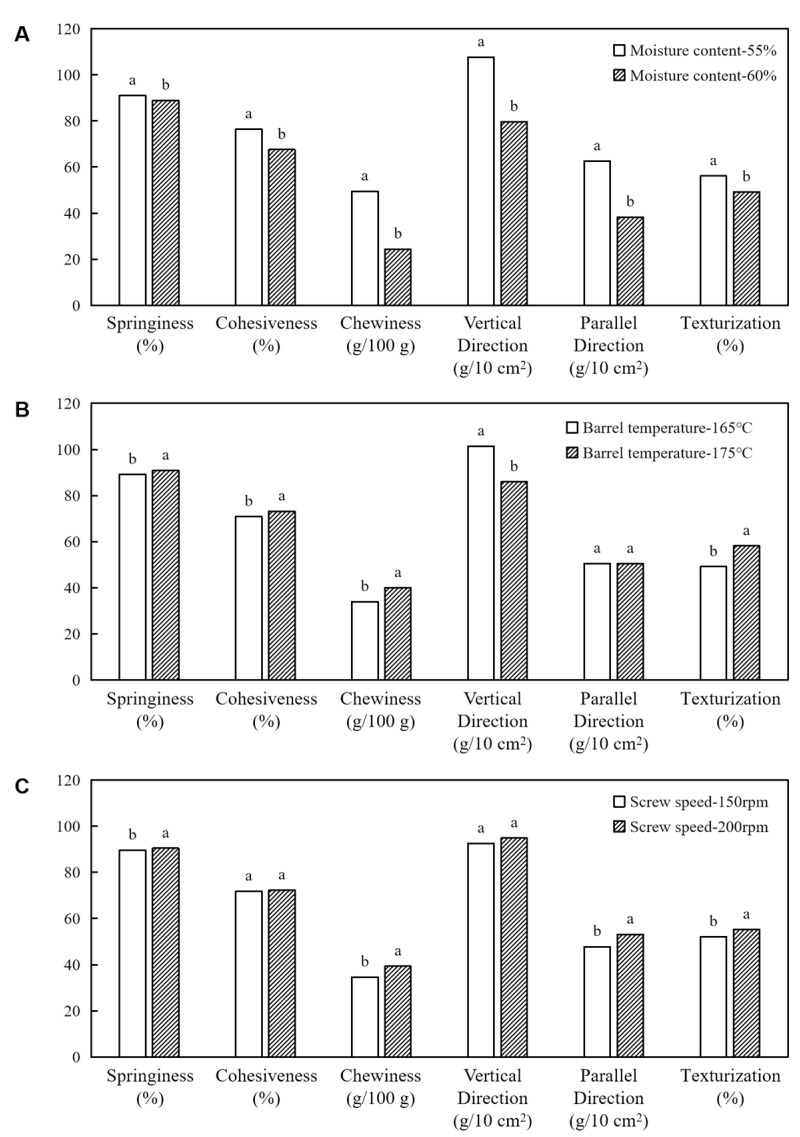
Average value of textural properties (springiness, cohesiveness, and chewiness), cutting strength (vertical direction and parallel direction), and texturization degree influenced by moisture content (**A**), barrel temperature (**B**), and screw speed (**C**). Bars with the same letter within the same textural property are not significantly different at *p* < 0.05 based on Duncan’s multiple range test.

According to Figure 5, a sharp decrease (*p* < 0.05) in all the tested parameters was observed with the increase in moisture content, possibly due to the soft texture of the product at high moisture contents [21,39,40]. This is consistent with the results of the ANOVA (Table 1); the moisture content had a highly significant (*p* < 0.001) effect on both the TPA and cutting strength. The barrel temperature and screw speed were positively correlated with the textural properties (TPA, cutting strength, and texturization degree) of the HMMA (Figure 5B,C). The reason might be attributed to a reduction in water in the barrel under high temperature and screw speed conditions that leads to protein aggregation, thereby increasing the textural properties of the HMMA after extrusion [39]. Wei et al. [37] found that the chewiness gradually decreased with increasing material moisture. Chen et al. [39] found that when the material moisture was increased from 28% to 60%, the degree of protein aggregation decreased, and the hardness and chewiness of the texturized soybean protein decreased significantly. Previous studies have shown that higher moisture contents and extrusion temperatures reduced the anisotropic index of a faba bean meat analog [36].

In addition, according to Table 1, the barrel temperature significantly affected (*p* < 0.05) the TPA, texturization, and vertical-direction cutting strength, but not that of the parallel direction. Zhang et al. [38] reported that, when the extrusion temperature was increased from 130 °C to 150 °C, the degree of disorganization gradually increased, indicating that the increase in extrusion temperature caused the material to fully melt, and protein–protein and protein–water interactions were enhanced. The screw speed only had a significant effect (*p* < 0.05) on the springiness, chewiness, texturization, and parallel-direction cutting strength. The softest HMMA texture was obtained in the present study when a moisture content of 60%, a barrel temperature of 165 °C, and a screw speed of 200 rpm were used. The hardest HMMA texture was obtained using a 55% moisture content, a 175 °C barrel temperature, and a 200 rpm screw speed (Table 2).

### 3.3. Structural Properties

#### 3.3.1. Macro- and Microstructure

**Figure 6:**
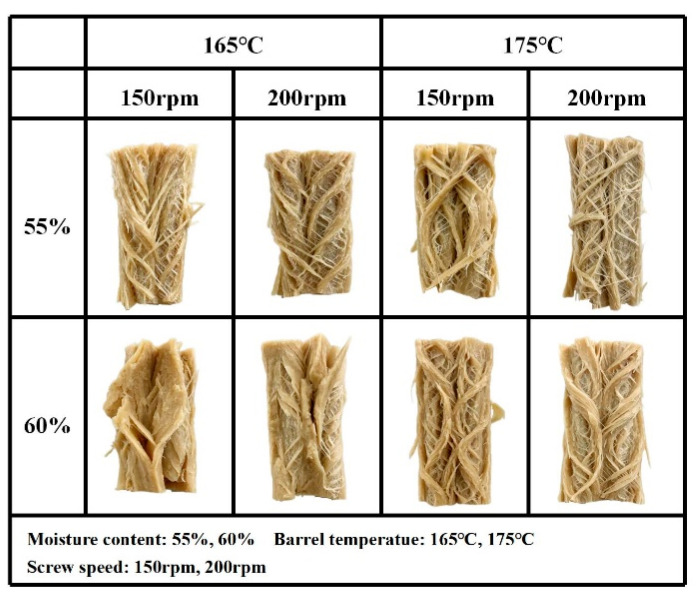
Macrostructure of meat analog with different process variables.

The macrostructure and microstructure of the HMMAs were visualized using a camera and SEM, respectively. The HMMAs possess dense, layered, and multiple fibrous structures, where long cooling die plays a key role in ensuring their formation. The fibrous structures of the HMMAs are depicted in Figure 6, where fibrillation was observed to increase at a low moisture content but a high barrel temperature and screw speed. Pietsch et al. [41] found that during the high-moisture extrusion of bran, the fibrous structure formed was easily destroyed when the extrusion temperature was 110 °C, whereas at a higher extrusion temperature, the extrudate had a rich fibrous structure. Combined with Table 2, the fiber structure of the meat analog is proportional to the TPA, cutting strength, and texturization. HMMAs with rich fiber structures are more elastic, chewier, and more texturized. In this study, the HMMA obtained from the extrusion condition of a 55% moisture content, 175 °C barrel temperature, and 200 rpm screw speed had the most abundant and dense fibrous structure.

**Figure 7:**
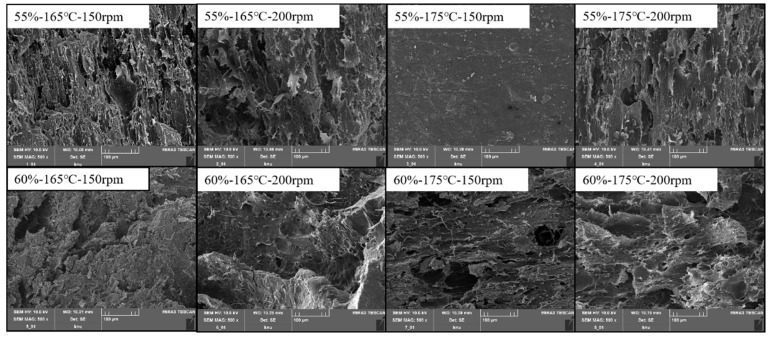
Scanning electron micrographs of meat analogs with different process variables (moisture content: 55 and 60%, barrel temperature: 165 and 175 °C, screw speed: 150 and 200 rpm).

The micrograph also revealed the uniform and compact fibrous microstructures of the HMMA when extruded at a low moisture content (55%) and high barrel temperature (175 °C) (Figure 7). The obtained results indicate the effectiveness of this condition for protein denaturation and texturization in HMMAs. The high degree of denaturation and reaggregation of the protein molecules at a low moisture content allowed the meat analog to produce a fibrous and reticulated structure like that of real meat. Li and Lee [42] pointed out that the changes in the microstructure of the meat analog were caused by changes in the chemical composition and protein interactions during the extrusion process.

#### 3.3.2. Secondary Structure

The peak intensity ratios of those spectra (Table 3) showed that the proteins in HMMAs and raw material blends mainly existed in the β-type structure, which has been reported as a stable protein conformation [35,43,44]. However, the β content in the HMMA (37.24–47.57%) was observed to be lower (*p* < 0.05) than that in the raw material blends (49.64%), which was consistent with the experimental results of Zhang et al. [35], suggesting a break in the chemical bond between protein molecules and the unfolding of the original conformation, leading to denaturation and subsequent aggregation [34,45]. The random coil content of the raw material blends was 19.16%, and it increased significantly (*p* < 0.05) after extrusion to a maximum value of 22.85% when a 55% moisture content was used to extrude the proteins at a 175 °C barrel temperature and a 150 rpm screw speed.

Heat and shear alter the conformation of proteins through the partial denaturation of the protein molecules, exposing groups that are normally concealed in folded native proteins, leading to an increase in disordered structures [46,47]. However, no significant difference (*p* > 0.05) in the random coil content among the HMMAs occurred, indicating that changes in the process variables had no significant effect in this regard. Jarpa-Parra et al. [48] reported that the presence of random coils promoted the formation of flexible protein structures and increased the adsorption of protein molecules at the interface. The α-helix content also increased significantly (*p* < 0.05) after extrusion, which is consistent with the results of Peng et al. [49]. In addition, they speculated that the percentage of each component of the protein secondary structure varies due to the raw material. The β-turns were significantly (*p* < 0.05) affected by the extrusion parameters, but there was no clear pattern. Beck et al. [50] proposed that the increase in the β-turn structure of pea protein after extrusion could be attributed to the formation of aggregates (intermolecular hydrogen bonds with β-turn structures).

## 4. Conclusions

In conclusion, in this study, IPP is considered a promising alternative to ISP for manufacturing raw materials for meat analogs. The moisture content is a crucial factor influencing protein denaturation and aggregation. A lower moisture content enhances the integrity index, TPA, and cutting strength of HMMAs while reducing the NSI, resulting in a dense fiber structure. The barrel temperature and screw speed only impact certain properties. The optimal conditions for producing IPP-based HMMAs were found to be a moisture content of 55%, a barrel temperature of 175 °C, and a screw speed of 200 rpm. These findings contribute to advancing the development and commercialization of subsequent meat analog products and establish a solid foundation for producing non-GMO, low-allergenicity IPP-based meat analogs. Future efforts should focus on enhancing IPP-based meat analog quality, such as through the addition of fat, the injection of carbon dioxide and nitrogen, and incorporating spices and nutritional supplements.

## Figures and Tables

**Table 1:** The effects of process variables and interactions on textural and physicochemical properties.

	Integrity Index (g kg^−1^)	Nitrogen Solubility Index (g kg^−1^)	Texture Profile Analysis			Cutting Strength (g cm^−2^)	
			Springiness (%)	Cohesiveness (%)	Chewiness (kg)	Vertical Direction	Parallel Direction
Moisture content (A)	83.55 ***	34.515 ***	3.18 × 10 ***	8.90 × 10 ***	6.76 × 10^2^ ***	9.91 × 10 ***	3.53 × 10^2^ ***
Barrel temperature (B)	871.893 ***	0.093	2.30 × 10 ***	6.27 *	3.92 × 10 ***	3.00 × 10 ***	0.00
Screw speed (C)	620.353 ***	67.659 ***	5.81 *	3.23 × 10^−1^	2.38 × 10 ***	6.60 × 10^−1^	1.71 × 10 ***
A × B	26.867 ***	21.954 ***	6.29 **	1.59 × 10 ***	2.42 × 10 ***	9.70 × 10^−2^	7.40 **
A × C	1.281	4.087	8.33 × 10^−1^	2.13	5.66 × 10 ***	1.45 × 10 ***	1.81 × 10 ***
B × C	389.471 ***	1.495	4.17 × 10^−1^	5.16 *	9.59 **	1.32 × 10 **	9.79 **

In each column, * is significant at *p* < 0.05; ** is significant at *p* < 0.01; and *** is significant at *p* < 0.001.

**Table 2:** Effects of process variables on TPA and cutting strength of meat analogs.

Process Variables			TPA			Cutting Strength (g cm ^−2^)		
MC (%)	BT (°C)	SS (rpm)	Springiness (%)	Cohesiveness (%)	Chewiness (g)	Vertical Direction	Parallel Direction	Texturization (%)
55	165	150	90.44 ± 0.85 ^ab^	77.25 ± 2.41 ^a^	4.516.37 ± 545.34 ^c^	1122.13 ± 134.75 ^a^	629.69 ± 47.62 ^b^	56.16 ± 3.38 ^bc^
		200	90.74 ± 1.12 ^ab^	77.11 ± 2.99 ^a^	5251.18 ± 482.39 ^b^	1177.48 ± 95.38 ^a^	657.70 ± 48.35 ^ab^	55.86 ± 1.51 ^bc^
	175	150	91.03 ± 0.53 ^ab^	73.78 ± 1.79 ^ab^	4184.50 ± 462.09 ^c^	901.79 ± 147.07 ^b^	513.80 ± 56.14 ^c^	56.98 ± 3.73 ^b^
		200	91.82 ± 0.80 ^a^	77.76 ± 1.58 ^a^	5841.75 ± 525.09 ^a^	1106.54 ± 156.73 ^a^	703.00 ± 97.47 ^a^	63.53 ± 2.45 ^a^
60	165	150	87.12 ± 2.04 ^d^	66.02 ± 5.24 ^c^	2097.40 ± 171.94 ^e^	985.28 ± 123.42 ^b^	366.42 ± 22.40 ^d^	37.19 ± 3.07 ^e^
		200	88.11 ± 1.59 ^cd^	62.94 ± 3.12 ^c^	1705.61 ± 116.58 ^f^	770.91 ± 75.63 ^c^	364.60 ± 23.42 ^d^	47.29 ± 2.20 ^d^
	175	150	89.56 ± 2.37 ^c^	69.93 ± 4.38 ^b^	3039.21 ± 235.91 ^d^	692.00 ± 62.68 ^c^	401.15 ± 40.12 ^d^	57.97 ± 2.10 ^b^
		200	91.01 ± 1.37 ^ab^	71.32 ± 6.14 ^b^	2920.11 ± 270.18 ^d^	737.83 ± 57.06 ^c^	400.50 ± 38.92 ^d^	54.22 ± 2.27 ^c^

Different letters following the data in the same column mean significant differences (*p* < 0.05). Moisture content—MC, barrel temperature—BT, screw speed—SS.

**Table 3:** Effects of process variables on the secondary structure of the meat analog.

Process Variables			β-Sheet (%)	Random Coil (%)	α-Helix (%)	β-Turn (%)
MC (%)	BT (°C)	SS (rpm)				
Raw material			49.64 ± 0.10 ^a^	19.16 ± 0.11 ^b^	14.77 ± 0.20 ^d^	15.77 ± 0.17 ^bc^
55	165	150	45.67 ± 0.32b ^cd^	22.53 ± 0.20 ^a^	17.69 ± 0.11 ^ab^	14.32 ± 0.15 ^d^
		200	47.57 ± 0.22 ^b^	21.44 ± 0.08 ^a^	16.24 ± 0.09 ^c^	16.66 ± 0.12 ^b^
	175	150	45.10 ± 0.33 ^cd^	22.85 ± 0.21 ^a^	17.90 ± 0.15 ^ab^	14.35 ± 0.15 ^d^
		200	45.04 ± 0.32 ^cd^	22.84 ± 0.20 ^a^	17.82 ± 0.10 ^ab^	14.51 ± 0.21 ^d^
60	165	150	37.24 ± 0.08 ^e^	21.12 ± 0.18 ^a^	18.63 ± 0.21 ^a^	23.54 ± 0.76 ^a^
		200	46.44 ± 2.27 ^bc^	21.45 ± 1.82 ^a^	17.08 ± 1047 ^bc^	15.22 ± 0.85 ^cd^
	175	150	46.56 ± 2.26 ^bc^	21.41 ± 1.85 ^a^	17.42 ± 0.88 ^abc^	15.15 ± 0.87 ^cd^
		200	44.23 ± 1.21 ^d^	22.47 ± 1.53 ^a^	17.76 ± 1.31 ^ab^	12.74 ± 1.17 ^bc^

Different letters following the data in the same column mean significant differences (*p* < 0.05).

## Data Availability

Data are contained within the article.
